# Merging perspectives: genotype-directed molecular therapy for hereditary diffuse gastric cancer (HDGC) and E-cadherin–EGFR crosstalk

**DOI:** 10.1186/s40169-018-0184-7

**Published:** 2018-02-22

**Authors:** Dandan Li, Winifred Lo, Udo Rudloff

**Affiliations:** 1 Thoracic & Gastrointestinal Oncology Branch, National Cancer Institute, Bethesda, MD USA; 2 Department of Surgery, University of Cincinnati College of Medicine, Cincinnati, OH USA

**Keywords:** Hereditary diffuse gastric cancer (HDGC), Epidermal growth factor receptor (EGFR), E-Cadherin (CDH1), E-Cadherin/catenin–EGFR cross-talk, Pharmacological vulnerabilities

## Abstract

Hereditary diffuse gastric cancer is a cancer predisposition syndrome associated with germline mutations of the E-cadherin gene (CDH1; NM_004360). Male CDH1 germline mutation carriers have by the age of 80 years an estimated 70% cumulative incidence of gastric cancer, females of 56% for gastric and of 42% for lobular breast cancer. Metastatic HDGC has a poor prognosis which is worse than for sporadic gastric cancer. To date, there have been no treatment options described tailored to this molecular subtype of gastric cancer. Here we review recent differential drug screening and gene expression results in c.1380del CDH1-mutant HDGC cells which identified drug classes targeting PI3K (phosphoinositide 3-kinase), MEK (mitogen-activated protein kinase), FAK (focal adhesion kinase), PKC (protein kinase C), and TOPO2 (topoisomerase II) as selectively more effective in cells with defective CDH1 function. ERK1-ERK2 (extracellular signal regulated kinase) signaling measured as top enriched network in c.1380delA CDH1-mutant cells. We compared these findings to synthetic lethality and pharmacological screening results in isogenic CDH1^−/−^ MCF10A mammary epithelial cells with and without CDH1 expression and current knowledge of E-cadherin/catenin–EGFR cross-talk, and suggest different rationales how loss of E-cadherin function activates PI3K, mTOR, EGFR, or FAK signaling. These leads represent molecularly selected treatment options tailored to the treatment of CDH1-deficient familial gastric cancer.

## Background

Hereditary diffuse gastric cancer (HDGC) is a cancer predisposition syndrome which accounts for up to 19–40% of familial gastric cancers and is associated with an autosomal-dominant inheritance pattern due to germline CDH1 variants [1–3]. While initially a syndrome used to describe familial inheritance of diffuse gastric cancer, it is now recognized that the syndrome includes increased risk for lobular breast cancer (LBC), possibly colorectal cancer, and non-cancerous but significant conditions like cleft lip palate syndrome [2–8]. Lifetime cumulative risk for gastric cancer in male CDH1 mutation carriers is 70% by age 80; similar risk for female CDH1 mutation carriers is 56% for diffuse gastric cancer and 42% for LBC [3]. Overall, the majority of CDH1 germline variants are truncating CDH1 mutations, followed by missense variants, and variants affecting splice sites [3, 9]. While CDH1 variants have been reported to affect each of the 16 exons of the gene, there is a non-random distribution with some hotspots reported including the truncating mutations c.1003C>T, c.1212delC, c.1137G>A, 1792C>T, or 2398delC [3, 9].

With recent advances in the understanding of the syndrome’s natural history and genetics, detailed guidelines have been developed for genetic testing and preventative interventions via endoscopic surveillance, prophylactic gastrectomy, and breast imaging [2, 3, 10]. Despite these measures, effective systemic therapies for patients who develop HDGC malignancies remain elusive. Patients with metastatic HDGC typically receive the same, largely ineffective chemotherapies as patients with sporadic gastric cancer; however, HDGC patients experience inferior outcomes to sporadic gastric cancer or gastric cancer with non-pathogenic CDH1 mutations [10, 11]. Thus, there remains a need for identification and selection of effective systemic agents for this unique patient subpopulation.

## High-throughput drug screening in cell-based models of HDGC

As no effective systemic therapies are available for HDGC, an initial broad evaluation of potential drug targets is desirable. Our group conducted a differential high-throughput drug screen in gastric cancer cells derived from a stage IV HDGC patient with a truncating c.1380delA CDH1 germline mutation and gastric cancer cells derived from a liver lesion of a gastric cancer patient with wild type CDH1 [12]. The drug library utilized for screening was enriched for oncology compounds and contained multiple compounds per class to detect class effects. In addition, pathway enrichment was derived from differentially expressed gene set(s) (DEG) in hereditary c.1380delA HDGC cells in comparison to a panel of sporadic gastric cancer cell lines. c.1380delA CDH1-mutant cells were selectively sensitive to inhibition of the EGFR effectors PI3K, mTOR, MEK, c-Src, FAK, and TOPO2 inhibition. The drug phenotype overlapped with the top two signaling networks found enriched by MetaCore analysis in c.1380delA cells [12]. The highest-ranking network predicted to be enriched in c.1380delA cells included a number of signaling regulators of the enriched epidermal growth factor receptor signaling pathway or inositol triphosphate (IP3)/diacylglyercol (DAG) signaling which directly or indirectly overlapped with the drug phenotype of enhanced sensitivity against MEK, mTOR, FAK, or PKC activity anti-PKC, c-Src kinase, and FAK activity. Table 1 lists the drug classes with selective activity in gastric cancer cells with hereditary CDH1 mutation compared to sporadic gastric cancer cells.

**Table 1:** Drug sensitivities derived from in vitro models of HDGC

	CDH1 MCF10A (−/−) [13]	CDH1 MCF10A (−/−) [13]	c.1380delA CDH1 HDGC [12]	c.1380delA CDH1 HDGC [12]
	qHTS drug phenotype	Lethality by siRNA target or target ligand	qHTS drug phenotype	Target kinase in enriched top network
Drug class				
*PI3K inhibitor*	*Yes*	PIK3CA, PIK3CG, PIK3R5, PIK3CB, PIK3CD, PIK3C2B	*Yes*	No
AKT1	No	AKT1	No	No
*mTOR inhibitor*	*Yes*		*Yes*	Yes
EGFR and PDGFR family inhibitor	Yes	PDGFD, EGFR, ERBB3, NRG1	No	Yes
Src kinase inhibitor	Yes		No	Yes
FAK inhibitor	?		Yes	Yes
*ALK/ROS1-like tyrosine* *kinase inhibitor*	*Yes*	ROS1, ALK	*Yes*	No
JAK family inhibitor	Yes	JAK2	No	No
BCL2 inhibitor	Yes	BCL2	No	No
*Aurora kinase inhibitor*	*Yes*		*Yes*	No
HDAC inhibitor	Yes	HDAC3, HDAC9, SIN3A, RERE	No	
ROCK inhibitor	No		Yes	No
Protein kinase C inhibitor	No		Yes	Yes

Quantitative high-throughput drug screening of MCF10A mammary epithelial cells vs isogenic CDH1^−/−^ MCF10A cells and hereditary c.1380del CDH1 gastric cancer cells vs sporadic CDH1 wild type SB.msgc-1 cells. Listed are vulnerabilities selective in CDH1^−/−^-mutant MCF10A and c.1380del CDH1 cells, shared drug classes are highlighted in italics

Active in both c.1380delA CDH1 mutant hereditary SB.mhdgc-1 and control CDH1 wild type SB.msgc-1 gastric cancer cells

Sensitivity to PI3K, mTOR, and EGFR inhibition was independently observed in another sentinel report on this subject conducted in MCF10A cells, a non-tumorigenic mammary epithelial cell line [13]. Telford et al. performed a broad, comparative genome-wide siRNA screen of isogenic MCF10A cells with and without CDH1 expression. G-Protein-coupled receptor (GPCR) signaling proteins and cytoskeletal proteins were selectively lethal upon siRNA-mediated knockdown in the CDH1^−/−^ null MCF10A cells. These genetic vulnerabilities overlapped with selective drug response profiles derived from a 4057 drug screen in the CDH1 isogenic MCF10A cell lines. Drugs and drug classes with increased sensitivity in the CDH1^−/−^ null MCF10A compared to CDH1 wild type cells included HDAC, PI3K, mTOR, JAK, BCL2, or aurora kinase inhibitors. Thus, when aligning drug phenotypes derived from both in vitro models of HDGC, CDH1^−/−^ null MCF10A and c.1380delA CDH1 HDGC cells, gastric cancer cells with defective CDH1 function showed selective overlapping sensitivity to PI3K, mTOR, ALK/ROS-1 like tyrosine, and aurora kinase inhibition in both systems. Table 1 list selective genetic and pharmacological vulnerabilities in CDH1^−/−^ null MCF10A and drug phenotype and enriched gene expression aberrations in c.1380delA HDGC cells. Differences in lineage and evolvement of screened cells, like pre-neoplastic primary mammary epithelial MCF10A cells versus metastatic cells derived from the ascites of a CDH1 germline mutation carrier with stage IV gastric cancer, or different coverages of the used drug libraries, might explain differences in observed drug phenotype like lack of HDAC inhibition, anti-Bcl2, and anti-XIAP sensitivity in the c.1380delA CDH1 cells or lack of sensitivity to MEK inhibition in the CDH1^−/−^ null MCF10A cells.

Indications of potential vulnerability to PI3KmTOR, or FAK inhibition are, in part, also corroborated by tissue studies on early T1a stage and > T2 lesions from CDH1-mutation carriers. Detailed pathology analysis of the early, non-proliferative intramucosal T1a lesions in prophylactic gastrectomy specimens of multiple family members with a c.1008G>T CDH1 germline mutation showed as the earliest, disease-initiating change reduced or absent expression of β-actin, p120 catenin, and Lin-7 compared to surrounding mucosa with general loss of organization of adherens junctions; [14]. Loss of adhesion function in the intramucosal stage was followed upon progression towards > T2 lesions by activation of c-Src kinase and FAK activation, and epithelial to mesenchymal transition (EMT). β-Catenin activation (as a result of p120 loss; measured by nuclear catenin staining) and mTOR activation (measured by staining with anti-phospho-mTOR Ser2448) was also observed in T1a lesions isolated in gastrectomy specimens of CDH1 mutation carriers with del124_126CCCinsT, c.521dupA, and c.1565+1G>A variants [9]. These observations of activation of the c-Src–FAK axis, catenin and mTOR signaling in clinical specimens appears to be in line with the drug phenotypes derived from the in vitro models.

## E-Cadherin/catenin–EGFR cross talk and potential mechanisms for pharmacologic targeting

**Fig. 1:**
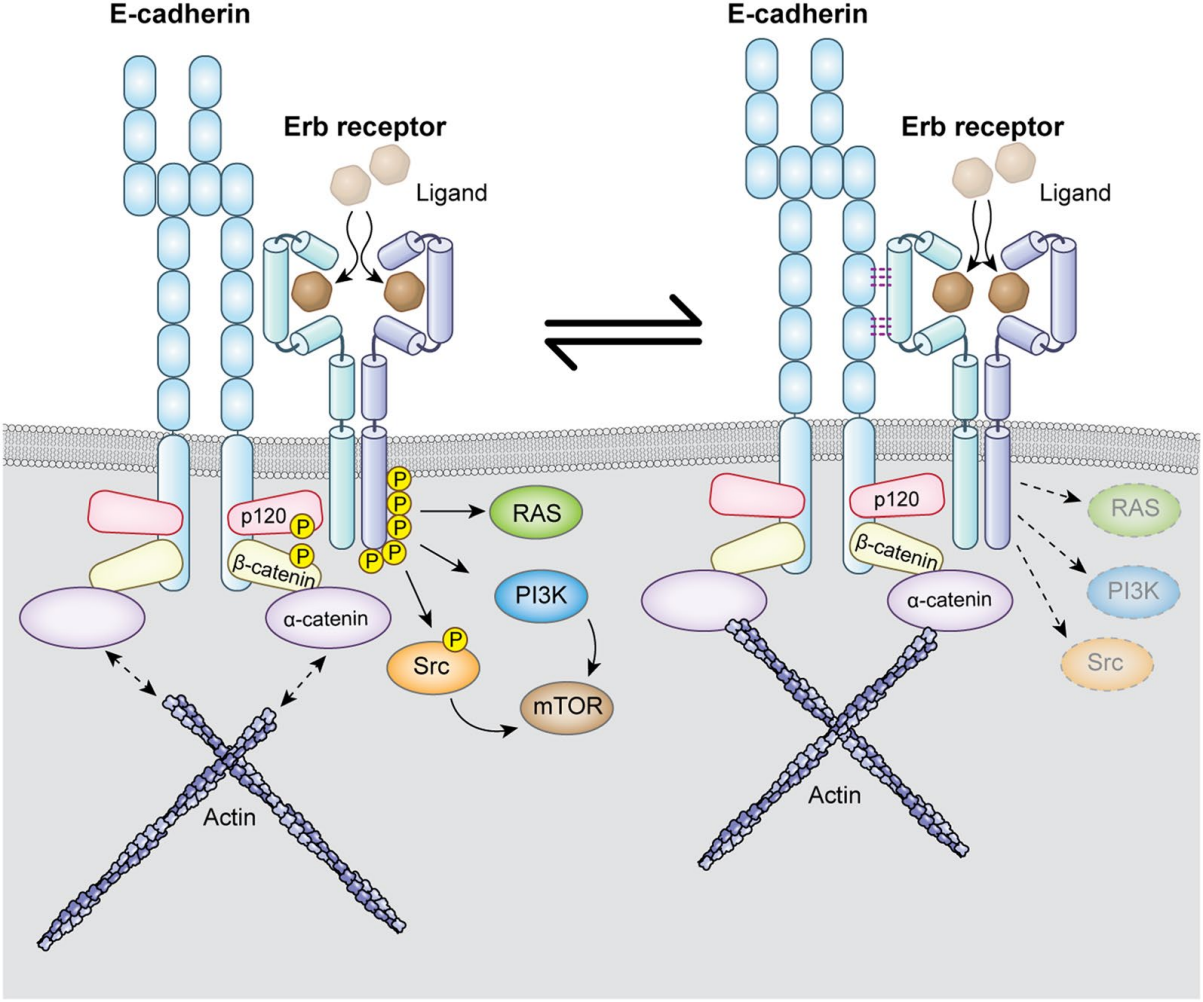
Bidirectional E-cadherin/catenin–EGFR cross talk in epithelial biology. Protein–protein interaction with the extracellular cadherin-binding domains of E-cadherin inhibits ligand-mediated activation of EGFR signaling (right); loss of E-cadherin–EGFR interaction leads to increased activation of PI3K, c-Src, and MAPK kinase pathway activation and phosphorylation and destabilization of the E-cadherin/catenin complex (left)

Early broad pharmacologic screens in in vitro models of diffuse gastric cancer indicate that dysregulated EGFR receptor and downstream effector signaling may be involved in aberrant signal transduction selective for CDH1-deficient gastric cancer cells. While c-Src kinase and FAK activation might be a direct result of elevated GPCR signaling, activation of ERK signaling in addition to enhanced PI3K and mTOR sensitivity suggest activation of upstream receptor tyrosinase kinase signaling in HDGC cells. Thus, how is E-cadherin/catenin complex dysfunction able to activate EGFR signaling and how can loss of CDH1 function explain above signal perturbation and drug phenotype? E-Cadherin/catenin signaling has long been known a downstream effector of EGFR signaling [15–18]. Upon ligand activation, EGFR promotes loss of cellular adhesion and increased migration and invasion, among other mechanisms, through phosphorylation of E-cadherin bound β-catenin, plakoglobin (γ-catenin) and p120ctn (δ-catenin 1), leading to destabilization of the E-cadherin/catenin/actin complex (Fig. 1) [17, 19–21]. As suggested by observed co-localization and cooperativity of the EGFR and E-cadherin/catenin complexes in the cell membrane of epithelial cells, phosphorylated EGFR directly interacts with both β- and δ-catenins [22–25].

Recently, there is increased appreciation of reverse E-cadherin/catenin–EGFR cross-talk as part of a bidirectional signaling axis in cancer pathogenesis. Inhibition of ligand-activated EGFR signaling by E-cadherin is hereby dependent on the integrity of the extracellular domains of E-cadherin and independent of β-catenin or p120ctn binding [26]. CDH1 missense mutant cell lines derived from families with missense mutations in the extracellular domains of E-cadherin were correspondingly less able to suppress EGFR signaling than cell lines with wild type E-cadherin [27–29]. Similarly, deleting mutations (exons 8 and 9 of CDH1) affecting the extracellular cadherin-binding domains of E-cadherin show increased EGFR activation [30]. Hence, loss of suppression of EGFR signaling by lack of E-cadherin/catenin–EGFR interaction in HDGC families with CDH1 germline mutations might explain the increased sensitivity to EGFR and PI3K kinase inhibition in CDH1-deficient HDGC (Fig. 1).

The increased sensitivity to FAK inhibition, or to the c-Src kinase inhibitor saracatinib and the selective loss of viability upon GPCR knockdown in CDH1^−/−^ MCF10A mutant cells, might be explained by increased GPCR signaling. GPCR signaling directly activates c-Src, and increased GPCR signaling has been suggested by elevated intracellular phosphatidylinositol 4,5-bisphosphate (PIP2) and phosphatidylinositol 3,4,5-trisphosphate (PIP3) levels (second messenger intermediates of GPCR signaling) in c.del1380 CHH1 HDGC cells. Of note, activation of the c-Src kinase and FAK system was inferred after loss of adherens function including reduced levels of actin, p120ctn, or Lin in the progression of intramucosal T1a lesions in CDH1 germline mutation carriers [14]. Loss of p120ctn is a pro-tumorigenic driver event in epithelial cancers with augmentation of EGFR signaling in breast cancer, elevated levels and activation states of c-Src kinase and FAK have been found to be associated with accelerated progression and shorter survival in epithelial malignancies including gastric cancer [31–33]. Inhibition of the Src kinase–FAK pathway can restore cell adhesion, reduce cell migration, and promote an epithelial phenotype [34].

**Fig. 2:**
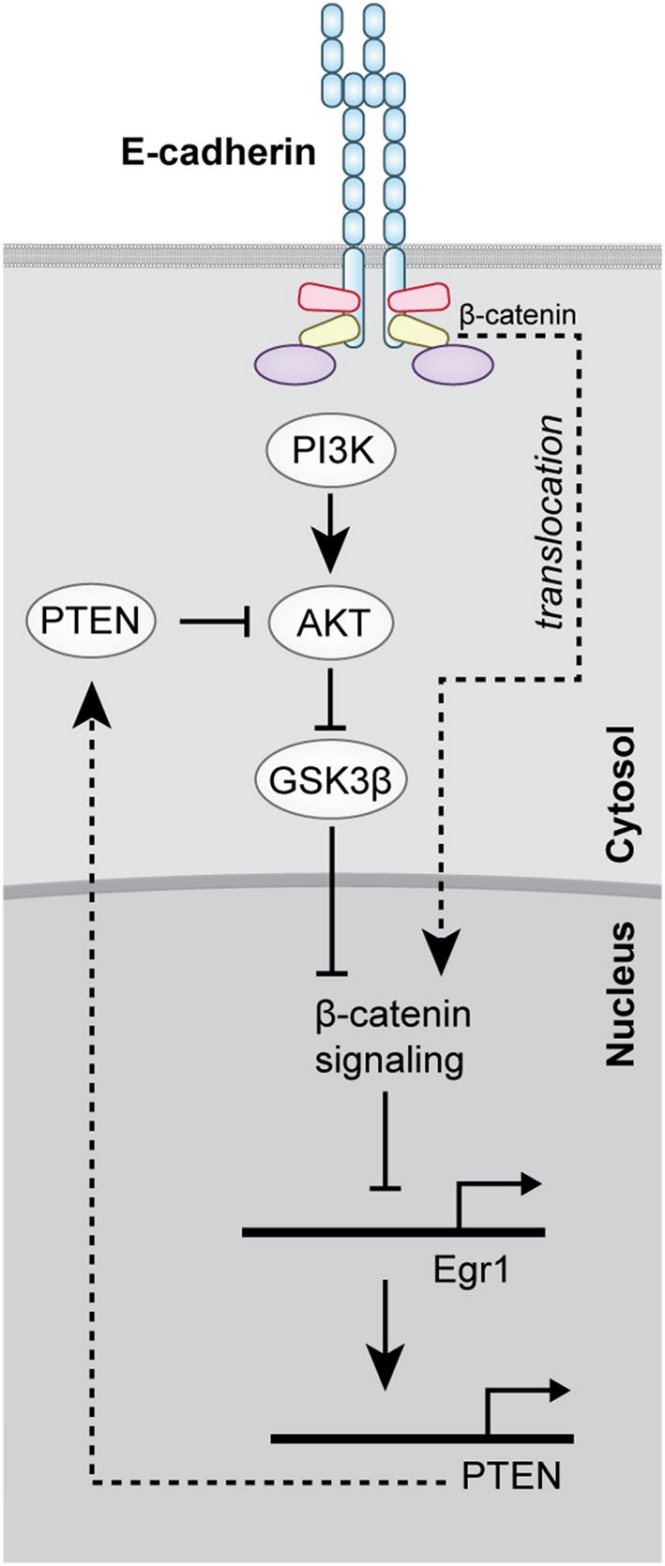
E-Cadherin/catenin–PI3K/AKT crosstalk. Release of negative feedback inhibition of PI3K/AKT signaling via reduced PTEN expression through elevated nuclear β-catenin levels. Disruption of the E-cadherin/catenin complex induces nuclear translocation of β-catenin repressing Egr-1-mediated PTEN expression leading to increased AKT activation

Activation of the EGFR downstream PI3K-mTOR pathway in CDH1-mutant cells may also be caused by the disruption of a negative feedback group of β-catenin and PTEN [35]. E-Cadherin loss is associated with enhanced nuclear β-catenin translocation, suppression of nuclear expression of EGR-1 and PTEN, and increased cytoplasmatic PI3K-AKT signaling promoting tumor cell growth (Fig. 2). A similar reciprocal relation of reduced E-cadherin expression levels and increased PI3K-AKT activation was seen in T1a lesions in prophylactic gastrectomy specimens from CDH1-mutation carriers; T1a lesions from three out of four CDH1 mutation carriers from different HDGC families with different truncating CDH1 mutations harbored activation of mTOR (measured by staining with anti-phospho-mTOR Ser2448) and catenin (measured by increased nuclear catenin staining) [9]. Thus, activation of PI3K-mTOR signaling in CDH1-deficient gastric cancer cells might be the result of multiple signal transduction aberrations including lack of suppression of ligand-mediated EGFR activation (Fig. 1) or lack of negative feedback inhibition of the PI3K–Akt axis via reduced PTEN levels (Fig. 2).

## Conclusions

Drug screening studies in isogenic CDH1^−/−^-mutant mammary epithelial MCF10A and c.1380delA CDH1-mutant gastric cancer cells show considerable overlap in sensitivity to PI3K, mTOR, or ALK/ROS-1 like tyrosine kinase inhibition. These pharmacological vulnerabilities are supported by comparative synthetic lethality, gene expression, and correlative tissue studies in clinical specimens of CDH1 mutation carriers, which indicate select perturbations of GPCR, actin-related, ERK1–ERK2, or FAK and c-Src kinase activity as signaling alterations and possible targets selective for CDH1-mutant gastric cancer cells. These pharmacological vulnerabilities are also supported by an improved understanding of the bidirectional cross-talk between E-cadherin/catenin and EGFR. E-Cadherin exerts direct and indirect negative regulation onto EGFR signaling, supporting blockade of the EGFR–PI3K kinase axis as therapy in this molecular subtype of gastric cancer. Considering that both anti-mTOR, anti-PI3K, and anti-EGFR therapies are already in routine clinical use or in late clinical development for a number of other cancer histologies, these observations suggest that PI3K and mTOR inhibitors may be considered for molecular-targeted therapies within clinical studies for patients with HDGC in the near future.

## Abbreviations

ALK: anaplastic lymphoma receptor tyrosine kinase
CDH1: E-cadherin
DAG: diacylglycerol
EGFR: epidermal growth factor receptor
EMT: epithelial–mesenchymal transition
ERK: extracellular signal regulated kinase
FAK: focal adhesion kinase
HDGC: hereditary diffuse gastric cancer
IP3: inositol trisphosphate
JAK: janus kinase
MEK: mitogen-activated protein kinase
mTOR: mammalian target of rapamycin
PI3K: phosphatidylinositol 3-kinase
PKC: protein kinase C
STAT3: signal transducer and activator of transcription 3
TOPO2: topoisomerase II
